# Targeting ovarian cancer and endothelium with an allosteric PTP4A3 phosphatase inhibitor

**DOI:** 10.18632/oncotarget.23787

**Published:** 2017-12-30

**Authors:** Kelley E. McQueeney, Joseph M. Salamoun, James C. Burnett, Nektarios Barabutis, Paula Pekic, Sophie L. Lewandowski, Danielle C. Llaneza, Robert Cornelison, Yunpeng Bai, Zhong-Yin Zhang, John D. Catravas, Charles N. Landen, Peter Wipf, John S. Lazo, Elizabeth R. Sharlow

**Affiliations:** ^1^ Department of Pharmacology, University of Virginia, Charlottesville, VA, USA; ^2^ Department of Chemistry, University of Pittsburgh, Pittsburgh, PA, USA; ^3^ Frank Reidy Center for Bioelectrics, Old Dominion University, Norfolk, VA, USA; ^4^ Department of Obstetrics and Gynecology, University of Virginia, Charlottesville, VA, USA; ^5^ Department of Medicinal Chemistry and Molecular Pharmacology, Purdue University, West Lafayette, IN, USA

**Keywords:** phosphatase, ovarian cancer, endothelium, inhibitor

## Abstract

Overexpression of protein tyrosine phosphatase PTP4A oncoproteins is common in many human cancers and is associated with poor patient prognosis and survival. We observed elevated levels of PTP4A3 phosphatase in 79% of human ovarian tumor samples, with significant overexpression in tumor endothelium and pericytes. Furthermore, PTP4A phosphatases appear to regulate several key malignant processes, such as invasion, migration, and angiogenesis, suggesting a pivotal regulatory role in cancer and endothelial signaling pathways. While phosphatases are attractive therapeutic targets, they have been poorly investigated because of a lack of potent and selective chemical probes. In this study, we disclose that a potent, selective, reversible, and noncompetitive PTP4A inhibitor, JMS-053, markedly enhanced microvascular barrier function after exposure of endothelial cells to vascular endothelial growth factor or lipopolysaccharide. JMS-053 also blocked the concomitant increase in RhoA activation and loss of Rac1. In human ovarian cancer cells, JMS-053 impeded migration, disrupted spheroid growth, and decreased RhoA activity. Importantly, JMS-053 displayed anticancer activity in a murine xenograft model of drug resistant human ovarian cancer. These data demonstrate that PTP4A phosphatases can be targeted in both endothelial and ovarian cancer cells, and confirm that RhoA signaling cascades are regulated by the PTP4A family.

## INTRODUCTION

Aberrant intracellular signaling is a recurrent feature of human cancers that is often reflected in substantial changes in pathways involving protein phosphorylation and dephosphorylation. Most protein tyrosine kinases are responsive to extracellular signals, but they are frequently mutated or dysregulated, promoting uncontrolled proliferation and enhanced cell survival. Despite substantial advances in the development of kinase-targeted therapeutics, these agents have thus far had little impact on ovarian cancer (OvCa) patient survival, which remains stubbornly at ∼45% five years after diagnosis. OvCa tumors are genetically complex and lack common molecular drivers [1]. Since protein tyrosine phosphatases (PTPs) appear to have a major role in the malignancy of cancer [2], we have initiated a program to develop small molecule probes to explore new therapeutic opportunities.

Studies with patient-derived tumor samples indicate a high level of expression of protein-tyrosine phosphatase 4A (PTP4A, also known as PRL) family members, and particularly PTP4A3 in ovarian cancers [3-6]. Elevated PTP4A3 expression has also been correlated with poor patient prognosis and metastatic processes in several cancer types [6-8]. While definitive endogenous PTP4A substrates have yet to be identified, genetic studies clearly implicate PTP4A in important cancer-associated signaling processes, thereby supporting a pivotal role for this PTP family in tumorigenesis [9-13]. Excess PTP4A promotes RhoA activation through a non-canonical interaction with the Src homology 3 domain of p115 RhoGTPase-activation protein [9]. PTP4A3 is highly expressed in the tumor-associated endothelium [3, 14], which has a central role in metastases formation and angiogenesis [12]. The altered microenvironment in tumors, including high concentrations of the potent vasodilator VEGF, creates a dysfunctional endothelium, which loses critical barrier properties. VEGF promotes the transcription of PTP4A3 through Myocyte Enhancer Factor 2C [15], and it has been documented that the loss of PTP4A3 attenuates VEGF-mediated vascular permeability *in vivo* [13]. The formation of OvCa peritoneal ascites is driven by vasodilation and loss of vascular membrane integrity, which enables tumor cell dissemination [13, 16]. Therefore, PTP4A3 phosphatase appears to influence the function of both tumor and stromal cells.

The interrogation of PTP4A3’s biochemical function and ability to serve as a therapeutic target would be greatly facilitated by potent and selective small molecule inhibitors. Although several candidates have been studied, including pentamidine, ginkgetin, sciadopitysin, emodin, BR-1, and some cyano-2-ene esters, most are neither potent (*i.e.*, IC_50_>25 µM) nor specific for PTP4A3. Current inhibitors are also frequently encumbered by structural liabilities, such as redox, metal-chelating, and electrophilic properties (reviewed in [17]). A thienopyridone was previously found to be a sub-micromolar PTP4A3 inhibitor (*in vitro* IC_50_∼150 nM) with considerable selectivity against 11 other phosphatases [18]. This compound suppressed anchorage-independent cancer cell growth in soft agar [18], but there is no available information about its *in vivo* activity. Our group recently designed and synthesized a novel iminothienopyridinedione, JMS-053, which proved to be a potent PTP4A3 inhibitor (*in vitro* IC_50_∼20 nM) [19]. Herein, the biochemical properties of JMS-053, including the inhibition of OvCa cell migration and spheroid growth, and the attenuation of *in vivo* tumor growth, are reported. Moreover, the discovery of a new role for PTP4A3 in controlling microvascular endothelial barrier function was also identified. Finally, JMS-053 served as a critical tool compound in identifying RhoA functions as a distal signaling convergence point for PTP4A activity in both endothelial and tumor cells.

## RESULTS

### *Ptp4a3* is amplified in serous cystadenocarcinoma and highly expressed in OvCa tumors

**Figure 1:**
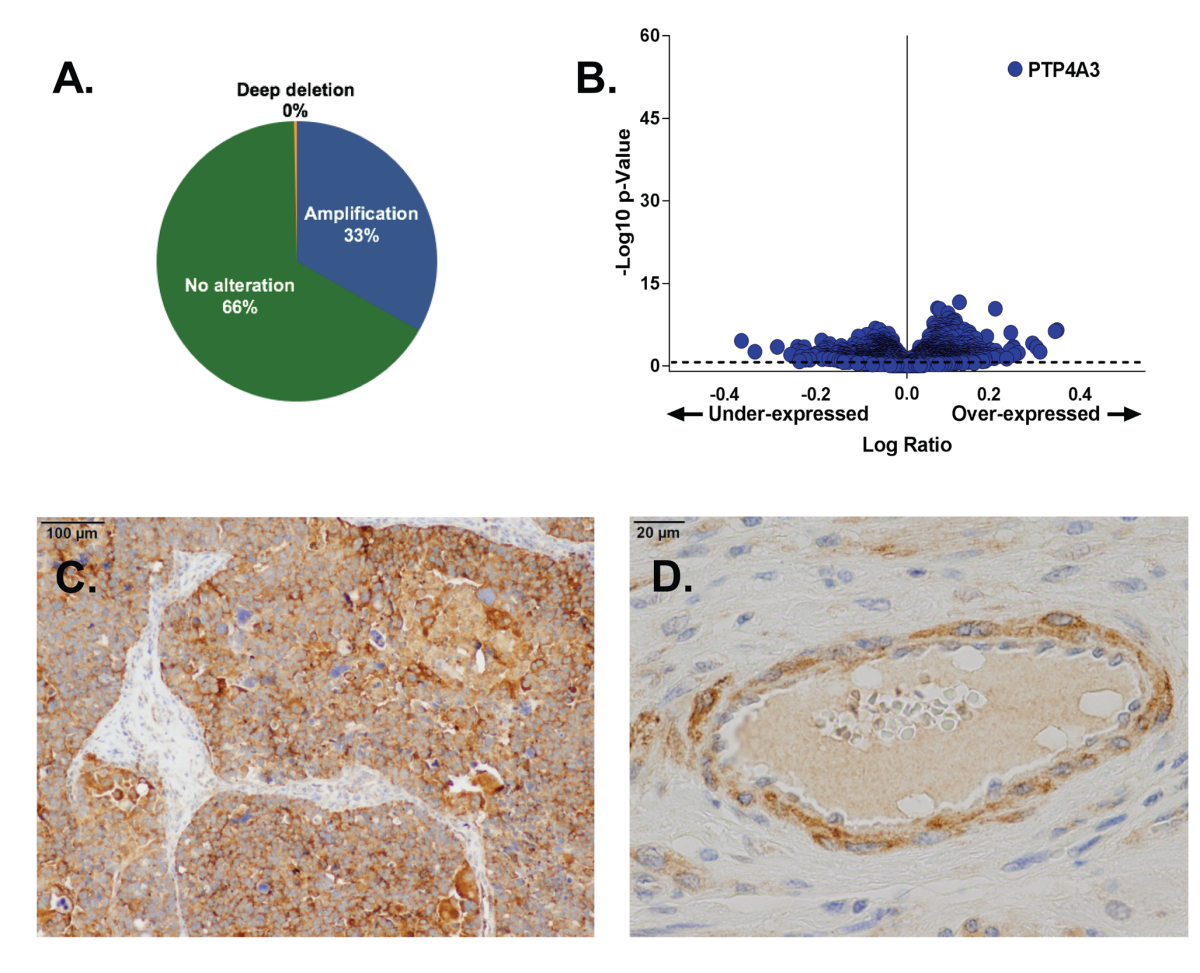
PTP4A3 expression is elevated in OvCa tumors Panel **A.** The *Ptp4a3* gene was amplified in 33% of the tumors of OvCa patients found in the TCGA Provisional database (594 total patients). Panel **B.** PTP4A3 mRNA was the most abundant mRNA in TCGA OvCa tumors as determined by RNAseq (307 OvCa patients). cBioPortal computes the relative expression of an individual gene in the tumor to the gene’s expression distribution in a reference population. The reference population is defined as all samples that are diploid for the gene in question or all profiled samples. Panel **C.** Immunohistochemical staining of OvCa patient tumor tissue showed enhanced PTP4A3 protein expression. Panel **D.** Enhanced PTP4A3 protein expression of PTP4A3 was seen in OvCa vascular endothelial cells and pericytes.

*Ptp4a3* is located in chr8q24, which is one of the most commonly amplified chromosomal regions in human cancers, including OvCa, suggesting a fundamental role in tumor maintenance or dissemination [20]. Previous studies of PTP4A3 expression profiles in OvCa used small sample sizes. Therefore, the most recent TCGA database from 594 patients (September 2017) (https://tcga-data.nci.nih.gov/tcga/) was analyzed, and *Ptp4a3* amplification in ∼33% of the ovarian serous cystadenocarcinomas (Figure 1A) and exceptionally high mRNA expression compared to the 13,860 genes examined in the companion RNASeqV2 data from 307 TCGA OvCa patients was confirmed (Figure 1B, Supplementary Figure 1A). Notably, PTP4A3 was rarely deleted or mutated. Because an increase in PTP4A1 or PTP4A2 mRNA was not observed, we subsequently focused primarily on PTP4A3 in OvCa.

Immunohistochemical analysis of human patient tumor samples from the University of Virginia Biorepository revealed PTP4A3 protein overexpression in 45/57 (79%) primary OvCa tumors (Table 1), as well as in the tumor endothelium and pericytes (Figure 1C-1D). These results provide further support that PTP4A3 overexpression occurs in human OvCa and add to its attractiveness as a candidate for pharmacological interrogation in both malignant and stromal populations within OvCa tumors.

**Table 1:** PTP4A3 protein expression in human patient samples as detected by immunohistochemistry

Cancer	PTP4A3 (Positive/Total)	Score			
		I	II	III	IV
High Grade Serous	41/52 (79%)	11	11	14	16
Endometrioid	2/2 (100%)	0	1	0	1
Mucinous	1/1 (100%)	0	0	1	0
Other	1/2 (50%)	1	1	0	0
Total	45/57 (79%)	12/57 (21%)	13/57 (23%)	15/57 (26%)	17/57 (30%)

### JMS-053 is a potent, specific, reversible, allosteric inhibitor of PTP4A3 phosphatase activity

**Figure 2:**
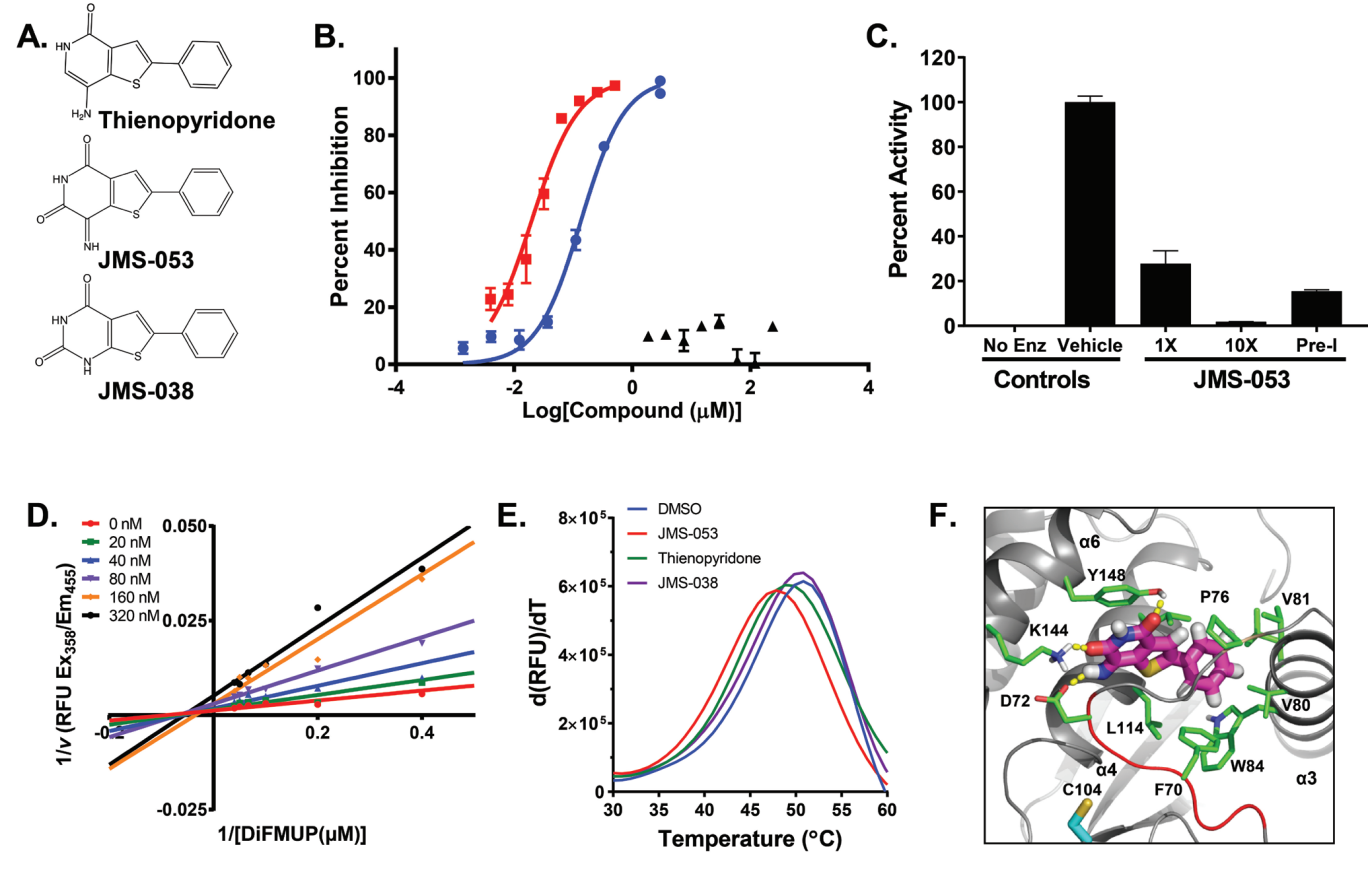
Structure, inhibitory activity, mechanism of action, and PTP4A binding of JMS-053 Panel **A.** Chemical structure of thienopyridone, JMS-053 and its inactive analog JMS-038. Panel **B.** PTP4A3 inhibition by JMS-053 using a full-length recombinant human PTP4A3 and DiFMUP as the substrate. 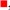, JMS-053; ▲, JMS-038; 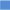, thienopyridone. Panel **C.** Reversible PTP4A3 inhibition by JMS-053. Full length PTP4A3 was treated with a concentration of JMS-053 equal to the IC_50_ or 10-fold higher, namely 18 or 180 nM, respectively. Pre-incubated (Pre-I) samples were exposed to a concentration of JMS-053 that was 10-fold higher than the IC_50_ for 30 min and then diluted to 18 nM, (IC_50_). Enz, enzyme. Panel **D.** Lineweaver Burk plot indicated noncompetitive inhibition by JMS-053. Panels B-D, Bars = SEM, *N* = 8. Panel **E.** Thermal shift assay for PTP4A3 protein (T_m_ = 50.5°C) showing destabilization by 100 µM JMS-053 (T_m_ = 47.5°C) and by 100 µM thienopyridone (T_m_ = 48.8^°^C). JMS-038 (100 µM) produced no significant thermal shift. Panel **F**. Proposed noncompetitive binding mode of JMS-053 (magenta carbons) with PTP4A3. Hydrogen bonds are shown with yellow dashes, and WPD loop residues W68PFDD72 are indicated by the red cartoon. For reference, active site residue C104 is shown with cyan carbons.

**Figure 3:**
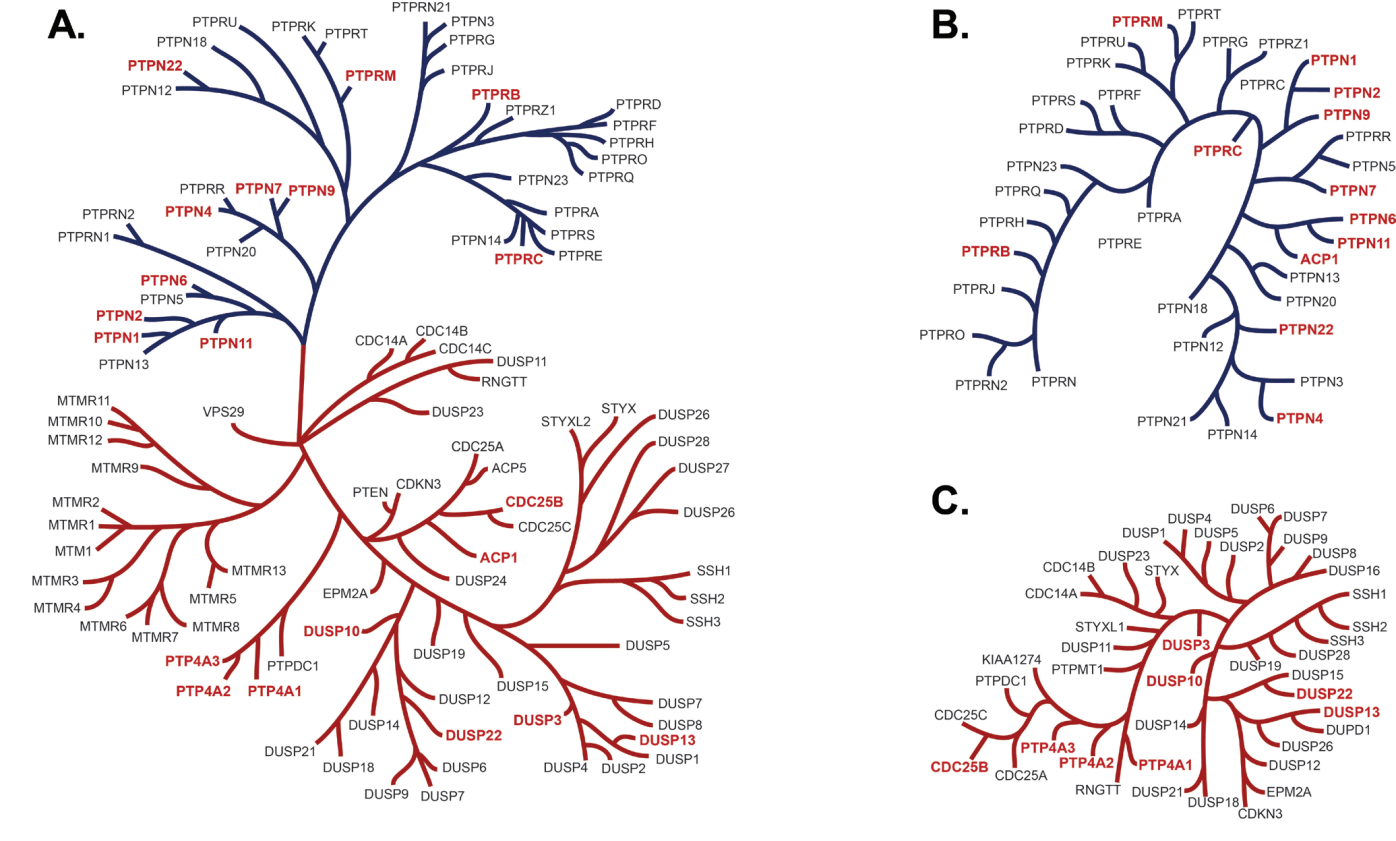
PTP dendrograms Panel **A.** PTP phylogenetic tree of the 14 amino acids in the active site as described by Almo *et al.* [49], depicting in red the PTPs tested for *in vitro* inhibition by JMS-053. JMS-053 inhibitory activity was tested on those phosphatases indicated in red. The blue branches indicate tyrosine phosphatases and the orange branches are the dual specificity phosphatases. Panel **B.** The phylogenetic tree represents the alignments of the longest isoform of the full length PTP as described by St-Denis *et al.* [50]. Marked in red are the PTPs that were assayed in this project. Panel **C**. The phylogenetic tree represents the alignments of the longest isoform of the full length dual specificity phosphatase with assayed phosphatases indicated in red.

The recently synthesized small molecule JMS-053 has an *in vitro* IC_50_ against human recombinant PTP4A3 of 18 nM, and currently is the most potent known inhibitor of this enzyme. It is a significant improvement over thienopyridone, which has an IC_50_ of ∼150 nM for PTP4A3 (Figure 2A-2B) [19]. A close structural analog of JMS-053, JMS-038, was inactive at concentrations as high as 100 µM, and therefore serves as a useful negative control compound (Figure 2A-2B). Furthermore, no structurally similar compounds with a 2D Tanimoto score of ≥0.8 were found during a search of the PubChem and ChemSpider databases, supporting the structural uniqueness of the JMS-053 chemotype. JMS-053 is a reversible PTP4A3 inhibitor, as indicated by dilution studies (Figure 2C). Reversible inhibition further distinguishes JMS-053 from many other PTP inhibitors, which frequently are irreversible, either because they form covalent adducts or generate species that oxidize the catalytic Cys [17]. Similar to thienopyridone, JMS-053 behaved as a noncompetitive inhibitor of the 6,8-difluoro-4-methyl-umbelliferyl phosphate (DiFMUP) substrate (Figure 2D. Supplementary Figure 1B). JMS-053 also had significantly improved *in silico* drug-like properties compared to the less active thienopyridone (Supplementary Table 1). Profiling JMS-053 against a panel of 25 other phosphatases (Figure 3A-3C, Table 2) and 50 kinases (Supplementary Table 2) showed that this chemotype was highly specific for the PTP4A family despite its rather small mass. The PTPs surveyed were broadly representative of the major human superfamily members, including three receptor-like PTPs, ten cytosolic PTPs, and five closely related dual-specificity phosphatases. This specificity was irrespective of whether one compared the amino acids in the catalytic domain (Figure 3A) or the full-length protein (Figure 3B-3C). JMS-053 also inhibited the two homologous PTP4A family members, PTP4A1 and PTP4A2, with IC_50_ values of 50 ± 14 and 53 ± 8 nM, respectively. Because both PTP4A1 and PTP4A2 also have oncogenic properties and are overexpressed in cancer, a pan-PTP4A family inhibition profile may be a valuable attribute for a small molecule therapeutic [5, 17]. Comparatively minimal inhibition was detected with the other phosphatases or kinases using a 1 µM screening concentration, with the exception of CDC25B and p38α, which were inhibited by ∼50%. Overall, JMS-053 showed at least a 20-fold selectivity for the PTP4A enzymes.

**Table 2:** Specificity profiling of JMS-053

Phosphatase	% Activity (Mean ± SD)
PTPRC (CD45)	88 ± 3
DUSP22	91 ± 6
PTPN7 (HePTP)	97 ± 3
ACP1 (LMPTP-A)	95 ± 4
LMPTP-B	99 ± 0
DUSP10 (MKP5)	87 ± 0
PTPN4 (PTP-MEG1)	100 ± 1
CDC25B*	52 ± 6
PTPN9 (PTP-MEG2)	88 ± 5
PTPN1 (PTP-1B)	90 ± 5
PTPN22	94 ± 2
PTPRB (PTPβ)	95 ± 5
PTPRM (RPTPμ)	80 ± 1
PTPN6 (SHP-1)	97 ± 11
PTPN11 (SHP-2)	100 ± 9
PTPN2 (TCPTP)	95 ± 3
DUSP13 (TMDP)	95 ± 1
DUSP3 (VHR)	87 ± 11
PTP4A1*	1 ± 0
PTP4A2*	0 ± 0
PTP4A3*	0 ± 0
PP1α	75 ± 0
PP2A*	99 ± 0
PP5	96 ± 2
YopH (Yersinia)	86 ± 4
Lambda PP (Phage)	72 ± 3

JMS-053 (1 µM) was profiled *in vitro* with 10 µM DiFMUP as a substrate against 26 phosphatases. Assays performed by a commercial vendor unless marked with an asterisk. N=2.

Based on kinetics studies, the K_i_ of JMS-053 for PTP4A3 was 18 nM. Initial attempts to co-crystalize JMS-053 with PTP4A3, however, have been unsuccessful. Therefore, PTP4A3 thermal denaturation profiling was conducted using SYPRO orange dye, an environmentally sensitive fluorophore that exhibits increased fluorescence on binding to hydrophobic protein segments. A *T*_*m*_ of 50.5 ± 0.6°C (*n* = 3) was obtained for the apo protein lacking the ligand, while adding JMS-053 (100 μM) produced a ∆*T*_*m*_ of 3.0°C or a destabilizing effect against PTP4A3 indicative of ligand binding [21] (Figure 2E). This ∆*T*_*m*_ exceeded the 1.7°C shift seen with a similar concentration of the much less potent thienopyridone. The negative control JMS-038 (100 μM) showed no effect on PTP4A3 stability. The recently available X-ray structure of PTP4A3 in a complex with CNNM3 (PDB 5TSR) was used to identify possible PTP4A3 binding modes for JMS-053 [22]. Based on computational docking studies with an energy refined structure, a putative binding site in the closed conformation was identified. In agreement with a noncompetitive mechanism (Figure 2D and 2F), the putative allosteric site is removed from the active site, and is located in a pocket that is flanked by the α3, α4, and α6 helices, and the WPD loop. As the highly flexible WPD loop clamps down on the active site, the imine moiety of the inhibitor can engage in a hydrogen bond with D72. As a result, this acidic residue would no longer be available to support enzymatic activity through interactions with catalytically important P-loop residues C104 and R110 [23]. Moreover, this tight, well desolvated inhibitor binding mode is predicted to hold the WPD loop in the closed conformation via: 1) hydrogen bonds between the compound’s pyridinedione oxygens and the side chain amine and hydroxyl groups of residues K144 and Y148, respectively, 2) π-stacking between the thienopyridinedione and the Y148 side chain aromatic ring, and 3) favorable π-π and hydrophobic contacts between the inhibitor’s phenyl substituent and the aromatic side chains of residues F70 and W84, as well as the alkyl side chains of residues P76, V80, V81, L85, L114, and L117, respectively. The proposed JMS-053 binding mode (Figure 2F) suggests that the thienopyridone structure is less active because it cannot engage in a hydrogen bond with K144, while JMS-038 is void of activity because it lacks the ability to orient in the proposed binding site such that a hydrogen bond with D72 is established. Notably, all of the proposed JMS-053 amino acids contacts, with the exception of V80, are conserved across all three PTP4A family members, which is consistent with this compound’s inhibitory profile.

### PTP4A3 inhibition normalizes trans-endothelial electrical resistance (TEER) after vascular endothelial growth factor (VEGF) or lipopolysaccharide (LPS) challenge

**Figure 4:**
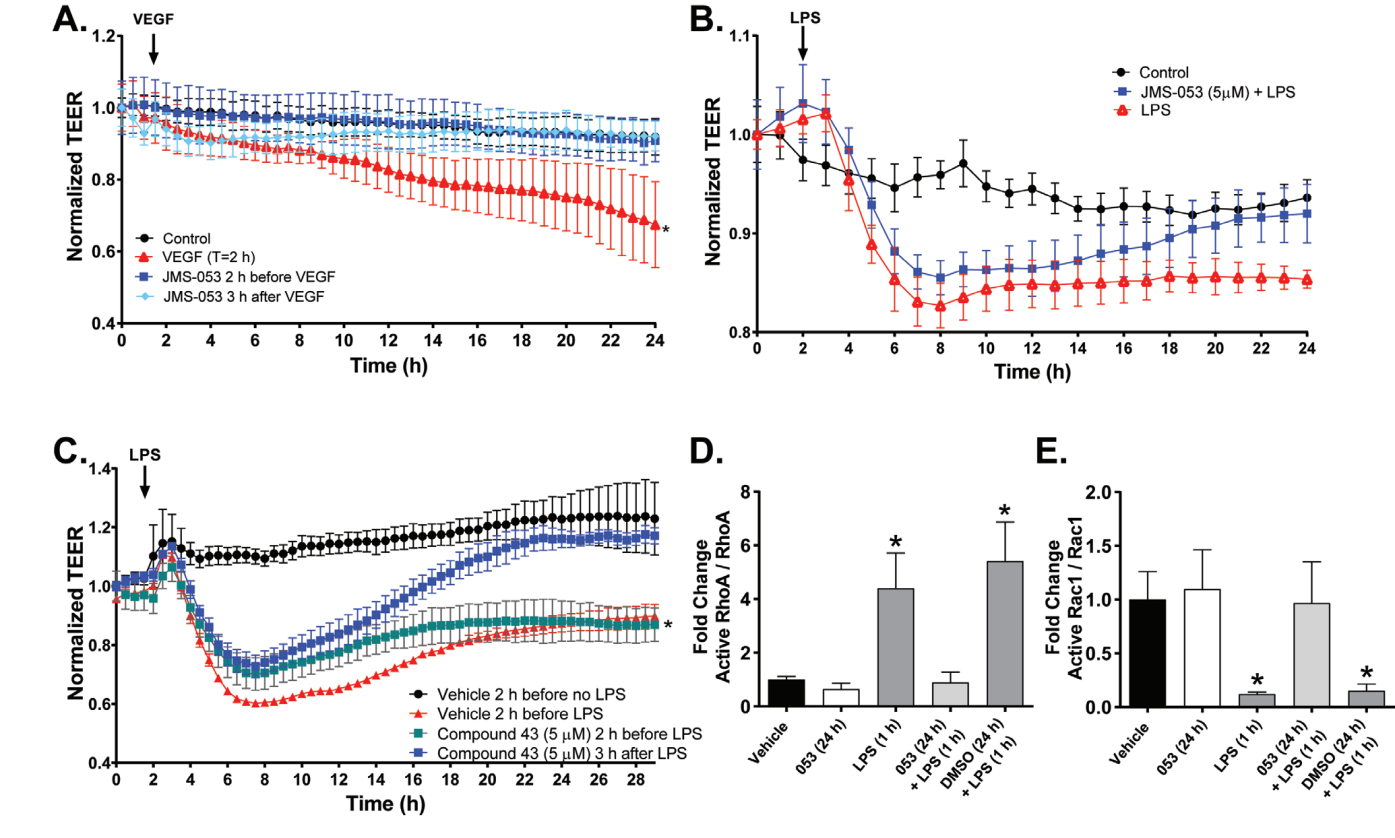
Pharmacological inhibition of PTP4A3 normalizes VEGF- and LPS-induced decreases in transendothelial electric resistance (TEER) Panel **A.** Confluent MVEC cells were treated with VEGF alone (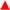), JMS-053 (5 μM) for 2 h before (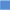) or 3 h after VEGF (100 ng/mL) (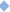) exposure (indicated by the black arrow). TEER was monitored for 24 h using ECIS. Resistance was normalized to time 0. Data are expressed as mean ± SEM, *N* = 3, *p < 0.05 *versus* vehicle control.● Panel **B.** Confluent MVEC cells were pretreated with (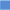) or without (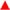) JMS-053 (5 μM) and 24 h later challenged with LPS (0.5 EU/mL) (LPS treatment indicated by the black arrow). Vehicle alone treated cells are indicated with the ● symbol. TEER was monitored over time using ECIS. Resistance was normalized to time 0. Data are expressed as mean ± SEM, *N* = 3, *p < 0.01 *versus* control. Panel **C.** Confluent MVEC were pretreated with 5 μM Compound 43 (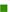) or vehicle control (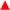) 2 h before 0.5 EU LPS (LPS treatment indicated by the black arrow) followed by continuous exposure to either Compound 43 or vehicle control or they were treated 3 h after LPS with 5 μM Compound 43 (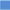). Vehicle alone treated cells are indicated with the ● symbol. TEER was monitored continuously using ECIS. Data are expressed as the mean ± SEM, *N* = 4. *p < 0.01 *versus* control. Panels **D.** and **E.** The effect of JMS-053 on the LPS induced RhoA activation and Rac1 deactivation by phosphorylation. Quantification of Western blot analysis of active RhoA, RhoA, active Rac1 and Rac1 in human MVEC pretreated for 24 h with vehicle (DMSO) or JMS-053 (5 µM) prior to treatment with vehicle or LPS treatment (0.5 EU/mL) for 1 h. Protein levels were normalized to total RhoA or Rac1. Signal intensity of active RhoA and Rac1 was quantified by densitometry. *p < 0.05 *versus* vehicle (two-way ANOVA). Data plotted as mean ± SEM. *N* = 3.

The vascular endothelium regulates macromolecular and cellular trafficking through the vessel walls. Enhanced vascular endothelial permeability is associated with OvCa pathogenesis and malignant ascites fluid formation, a key characteristic of OvCa that is often present at diagnosis [24]. Many substances increase trans-endothelial permeability including growth factors, such as VEGF, proinflammatory factors, such as cytokines, and exogenous substances like bacterial LPS. The loss of endothelial barrier function could be due, at least in part, to elevated levels of VEGF in the tumor microenvironment [25]. Because a marked *in vivo* reduction in VEGF-induced vascular permeability has been observed in mice lacking PTP4A3 [13], the impact of JMS-053 on the integrity of MVEC barrier function was evaluated. Barrier function was measured by differences in trans-endothelial electrical resistance (TEER), a method that has been repeatedly demonstrated to reflect changes in macromolecular permeability and allows for continuous, quantifiable, real-time monitoring of barrier function [26, 27]. Exposure of MVECs to VEGF (100 ng/mL) induced a profound disruption or “leakiness” of the endothelial barrier function that persisted for at least 22 h (Figure 4A). The precipitous decrease in endothelial barrier integrity after VEGF treatment was normalized when MVEC were pretreated for 2 h with 5 µM JMS-053 and then continuously exposed to the compound (Figure 4A). Post-treatment with JMS-053 at 3 h after VEGF exposure normalized endothelial barrier function, indicating the lack of a requirement for pretreatment (Figure 4A).

MVEC barrier dysfunction is also caused by LPS and is mediated by RhoA activation [28]. The loss of MVEC barrier is frequently caused by changes in the actin cytoskeleton where cortically distributed actin is re-arranged to form stress fibers via activation of RhoA and a decrease in Rac1. Exposure to JMS-053 24 h prior to LPS treatment prevented LPS-induced disruption of the endothelial barrier function (Figure 4B). There was no observed mitigation of LPS-induced vascular permeability with JMS-053 treatment 3 h after LPS exposure. Protection and mitigation of VEGF-induced increased endothelial permeability was also observed with a recently described inhibitor of PTP4A trimerization Compound 43 [7] (Figure 4C), which is structurally and mechanistically quite distinct from JMS-053. This finding provided strong orthogonal support for a role of the PTP4A family in the regulation of endothelial permeability. JMS-053 treatment blocked both the LPS-mediated RhoA activation and the LPS-mediated reduction in Rac1 activation (Figure 3D-3E). Collectively, these data suggest PTP4A phosphatases have a regulatory role in endothelial barrier function through the RhoA-Rac1 axis.

### JMS-053 inhibits OvCa proliferation, spheroid viability, and migration

**Figure 5:**
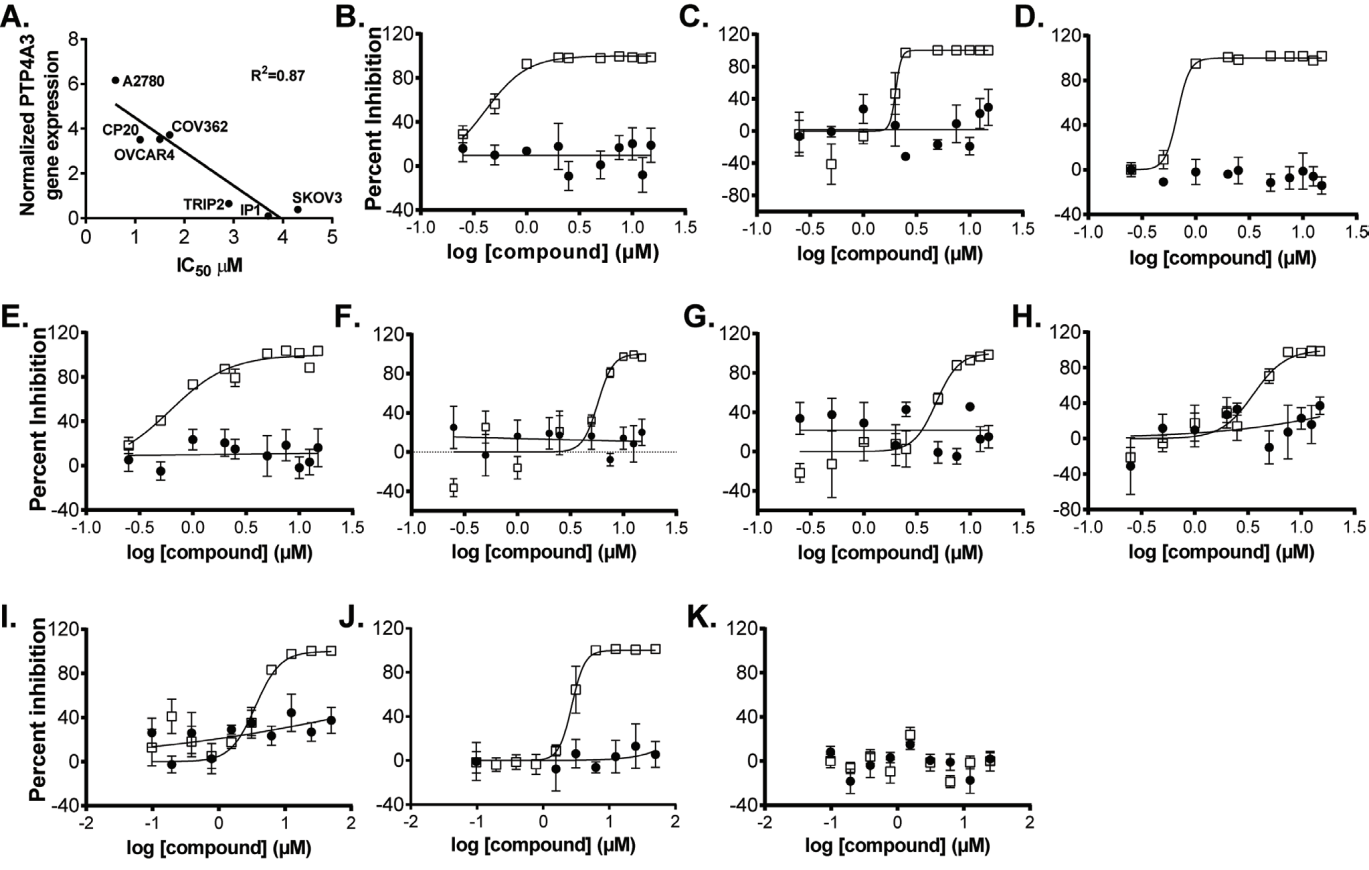
Effect of JMS-053 and control compound treatment on OvCa cell line and human normal ovarian epithelial cell spheroid growth Representative IC_50_ curves for JMS-053 (□) and control compound (●) JMS-038, a structurally related inactive JMS-053 analog. Panel **A.** Relationship between PTP4A3 mRNA expression and IC_50_ values for JMS-053; Panel **B.** HeyA8; Panel **C.** HeyA8-MDR; Panel **D.** A2780; Panel **E.** A2780CP20; Panel **F.** SKOV3; Panel **G.**, SKOV3IP1; Panel **H.** SKOV3TRIP2; Panel **I.** OVCAR4; Panel **J.** COV362: and Panel **K.** HI0-180 normal ovarian epithelial cells. Independent assays (*N* = 3) with *N* = 4 replicates.

The antiproliferative effects of JMS-053 were compared to the nonspecific PTP4A3 inhibitor BR-1 [29] and thienopyridone [18] using OvCa cells plated as a monolayer in cell culture microtiter plates and treated with compounds for a total of 48 h. The most sensitive cell line was A2780, with an IC_50_ for JMS-053 of 600 nM (Table 3). OvCa cells that are resistant to either cisplatin or paclitaxel remained sensitive to JMS-053. Moreover, JMS-053 was 30-44 fold more potent than BR-1 in inhibiting the growth of some of the OvCa cell lines and was on average 8-fold more potent that thienopyridone (Table 3). The negative control compound JMS-038 did not significantly inhibit the growth of any of the OvCa cell lines at concentrations as high as 50 µM. Response to JMS-053 was unrelated to the population doubling time as illustrated by the faster growing A2780CP20 cells, which have a doubling time of 19.3 h and an IC_50_ of 1.1 ± 0.7 µM, compared to the parental A2780 cells, which had population doubling time of 27.7 h and an IC_50_ of 0.6 ± 0.7 µM. JMS-053 did not inhibit the growth of IMR90 fibroblasts. We observed a correlation between the IC_50_ values and PTP4A3 expression (Figure 5A) but we have been unsuccessful in quantifying endogenous PTP4A3 protein levels in these cells. Others [30] have demonstrated posttranslational control of PTP4A3 protein. Moreover, PTP4A3 phosphatase activity is regulated by SRC-mediated phosphorylation on Y53 [31], which adds yet another level of complexity to the analysis.

**Table 3:** Effects of JMS-053 on OvCa monolayer proliferation and spheroid viability

Cell line	Monolayer (IC_50_ µM)					Spheroid (IC_50_ µM)	
	BR-1	Thieno-pyridone	JMS-053	JMS-038	Doubling time (h)^a^	JMS-053	JMS-038
SKOV3	48.3 ± 9.1	28.8 ± 2.2	4.3 ± 1.1	>50	43.3	10.5 ± 1.5	>50
SKOV3IP1	35.2 ± 2.3	31.3 ± 8.5	3.7 ± 0.4	>50	27.8	5.2 ± 0.7	>50
SKOV3TRIP2	46.5 ± 0.3	>50	2.9 ± 0.6	>50	35.8	5.5 ± 2.4	>50
A2780*	26.4 ± 1.0	4.5 ± 0.6	0.6 ± 0.2	>50	27.7	1.1 ± 0.5	>50
A2780CP20**	17.3 ± 2	13.1 ± 06	1.1 ± 0.04	>50	19.3	1.6 ± 0.7	>50
HEYA8*	>50	47 ± 4.9	25.2 ± 8.5	>50	19.0	0.8 ± 0.3	>50
HEYA8-MDR**	>50	>50	19.1 ± 6.0	>50	20.8	3.5 ± 1.4	>50
OVCAR4	48.5 ± 7.9	15.5 ± 2.4	1.5 ± 0.3	>50	39.8	4.4 ± 2.8	>50
COV362	ND_b_	ND	ND	ND	ND	1.6 ± 0.9	>50
IMR90	ND	ND	>50	>50	<24 h_c_	ND	ND
HI0-180	ND	ND	ND	ND	ND	>25	>25

Monolayer and spheroid cells were treated for 48 h with compounds. * cisplatin resistant; **, paclitaxel resistant. Mean ± SD µM. N=3.

^a^ One determination.

^b^ ND = not determined

^c^ From [43]

**Figure 6:**
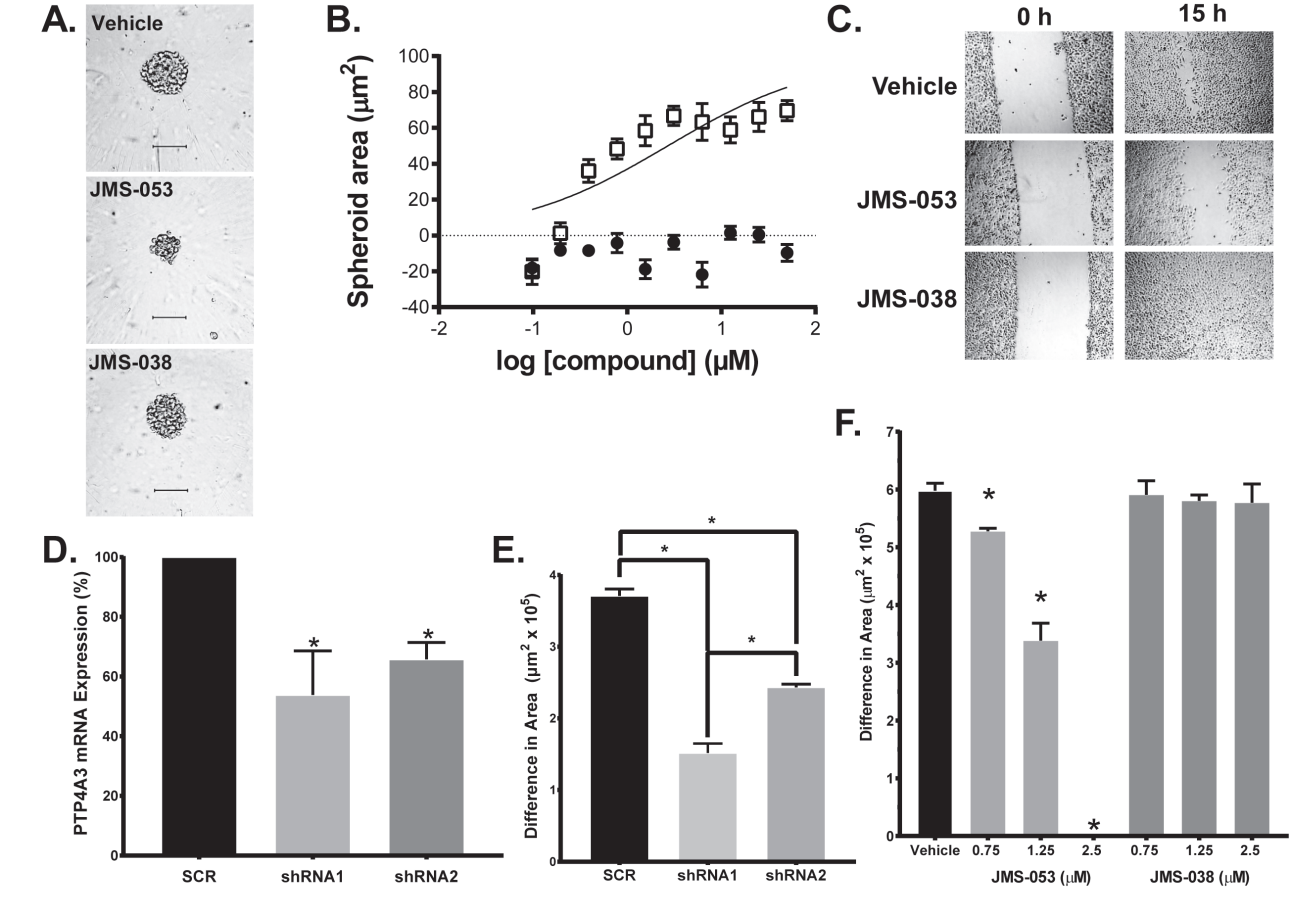
Genetic and pharmacological inhibition of PTP4A3 Panel **A.** JMS-053 but not JMS-038 inhibited HeyA8 cell spheroid growth. Images were taken of the spheroids 48 h after plating and 24 h after compound addition. Bar = 100 μm. Panel **B.** Quantification of JMS-053 effects on spheroid area. HeyA8 cells were incubated and treated with different concentrations of JMS-053 (□) or control compound JMS-038 (●). Brightfield spheroid images were captured 48 h after compound exposure and spheroid area was quantified using Harmony software. Panel **C.** Scratch wound assay. JMS-053 inhibited HeyA8 migration after wounding. Images of the cells were taken of the vehicle, JMS-053, and JMS-038 treated HeyA8 cell monolayers 0 and 15 h after the longitudinal scratch. Panel **D.** Suppression of PTP4A3 mRNA levels by shRNA. Treatment of HeyA8 cells with scrambled (SCR) or two different PTP4A3-targeted shRNA for 48 h decreased PTP4A3 mRNA, as measured by qRT-PCR. Panel **E.** Quantification of migration inhibition by shRNA. Confluent HeyA8 cells expressing shRNA were longitudinally wounded with a pipet tip and cell migration was measured after 14 h by determining the open area remaining from the scratch using multiple points of the cell front and ImageJ Fiji software. Panel **F.** Quantification of inhibition of cell migration by JMS-053. Confluent HeyA8 cells were longitudinally wounded with a pipet tip and cell migration was measured 14 h later as described above. JMS-053 but not JMS-038 blocked the migration of HeyA8 cells. *p < 0.05, *N* = 3.

Because OvCa often presents with ascites fluid containing cancer cell aggregates, JMS-053 was evaluated using relevant OvCa spheroids and a 48 h exposure. OvCa cell line spheroids adopted varied morphological features (Supplementary Figure 2). HeyA8 spheroids were most responsive to JMS-053 with an IC_50_ value of 0.8 µM, and the paclitaxel-resistant HeyA8-MDR cells retained sensitivity to JMS-053 with an IC_50_ of 3.5 µM (Figure 5B-5C, Figure 6A, Table 3). A2780, A2780CP20, SKOV3IP, SKOV3TRIP2, A2780, A2780CP20, COV362 and OVCAR4 cells had IC_50_ values between 1.1-5.5 µM (Figure 5D-5E, 5G-5J, Table 3, Supplementary Figure 3). The least responsive was the SKOV3 parental cell line with an IC_50_ value of 10.5 µM (Figure 5F, Table 3). JMS-053 did not reduce normal human ovarian HI0-180 epithelial cells survival at concentrations up to 25 µM, suggesting minimal toxicity at least against this non-tumorigenic cell population (Table 3, Figure 5K). A 48 h exposure to the control compound JMS-038 did not show any effects at concentrations up to 50 µM in any cell lines.

PTP4A3 overexpression promotes, and genetic deletion reduces, cancer cell migration [10, 13]. Consistent with this observation, JMS-053 significantly inhibited HeyA8 cell migration in a scratch wound assay at concentrations as low as 750 nM (Figure 6B-6C). Similarly, shRNA knockdown of PTP4A3 also diminished HeyA8 cell migration, supporting the specificity of the JMS-053-induced response (Figure 6D-6E). The substantial difference in cellular IC_50_ values *versus* the *in vitro* PTP4A3 IC_50_ value could be due to a combination of factors, including cell membrane permeability, intracellular compartmentalization, protein binding, and compound degradation.

### JMS-053 suppresses RhoA activation in OvCa cells

**Figure 7:**
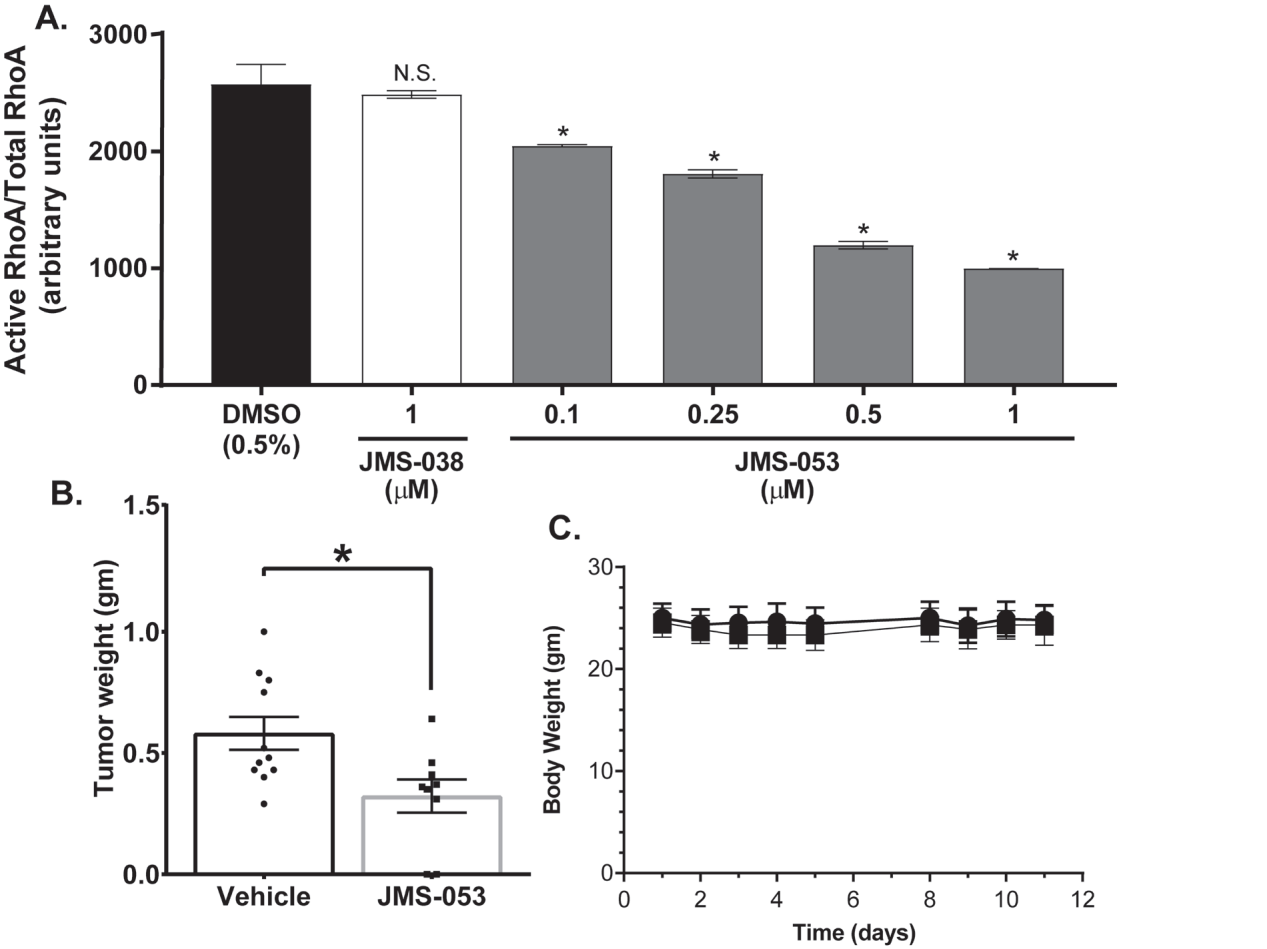
JMS-053 inhibits RhoA activation in HeyA8 cells and has antitumor activity Panel **A.** Cells were starved in serum-free RPMI 1640 for 24 h, then activation of RhoA was induced by exposure to 10% serum. Cells were pre-treated with indicated concentrations of compounds for 30 min prior to serum stimulation and for 30 min while being stimulated with medium containing 10% FBS. GTP bound RhoA in equal amounts of total lysate was quantified by a luminescence based G-LISA RhoA Activation assay. Treatment with 0.1-1 μM JMS-053. Constitutively active RhoA was used as a positive control for the binding of GTP-RhoA to G-LISA. The data were expressed as relative luminescence units (RLU) and were the mean ± SEM (*N* = 4). Statistical significance was determined by one-way ANOVA and Tukey’s multiple comparison test. *p < 0.05, n.s. = not significant. Panel **B.** Female, athymic nude mice with HeyA8-MDR tumors were injected IP with vehicle or JMS-053 (10 mg/kg) daily for 5 days followed by two days without treatment and four additional daily treatments (10 mg/kg). Error bars = SEM. *p < 0.05 (Mann-Whitney). Panel **C.** Body weight monitoring for 12 days indicates that JMS-053 treatment did not cause significant weight loss.

The phenotypic impact of genetic and pharmacologic PTP4A3 inhibition prompted us to investigate what changes in cell signaling drive these PTP4A3-dependent cellular behaviors. RhoA activation is known to be integral to the promotion of growth, migration, and dissemination of tumor cells [32], and elevated PTP4A3 expression levels are reported to alter RhoA activity [10, 12, 32]. Therefore, the impact of JMS-053 treatment on the activation of RhoA in HeyA8 cells was investigated. HeyA8 cells were serum-starved overnight and then pre-treated with JMS-053 (0.1-1 µM) or vehicle for 30 min followed by a 30 min exposure to medium supplemented with 10% FBS to stimulate RhoA activation in the presence of either JMS-053 or vehicle. JMS-053 treatment caused a significant inhibition of RhoA activation at all concentrations tested with an IC_50_ of 0.6 µM (Figure 7A). Treatment of HeyA8 cells with the control compound JMS-038 (1 µM) had no significant effect compared to the DMSO vehicle control. Treatment with PTP4A3 targeted shRNA reduced RhoA activation and JMS-053 (500 nM) caused no further reduction in RhoA activation consistent with PTP4A3 target engagement (Supplementary Figure 4).

### JMS-053 treatment inhibits drug-resistant OvCa tumor growth *in vivo*

Drug resistance remains a challenging aspect of OvCa treatment. Therefore, in a pilot study, the *in vivo* activity of JMS-053 was examined using a HeyA8-MDR nude mouse tumor model. Mice readily tolerated an IP dose of 10 mg/kg of JMS-053 and, based on an independent pharmacokinetic study by SAI Life Sciences, the maximum plasma concentration was reached within 30 min and plasma concentrations of 225 ng/mL (879 nM) were present 8 h after the injection. The area under the plasma concentration curve (AUC) was 4.425 h*µg/mL, which indicates a sustained presence of compound. Female nu/nu mice bearing IP HeyA8-MDR tumors were used, because the IP implantation site is very representative of the human disease [33]. Tumor bearing mice were treated with vehicle or 10 mg/kg JMS-053 over a nine-day period. IP OvCa tumors were observed in all vehicle treated mice and in 7 of 9 JMS-053 treated mice. The tumors from the mice treated with JMS-053 for nine days weighed on average 45% less than those from the vehicle treated group (Figure 7B). There was no significant difference in the body weights of the mice in the two treatment groups (Figure 7C), thereby indicating that JMS-053 was well tolerated at the doses that were administered.

## DISCUSSION

Epithelial OvCa is the most lethal gynecological cancer and, unfortunately, it is usually diagnosed at advanced stages when treatment strategies are limited. Despite an initial response to surgical debulking and platinum chemotherapy, recurrent or relapsed disease is common. Accumulation of ascites fluid enables the dissemination of OvCa cells or spheroid aggregates along the peritoneal cavity, a characteristic that differentiates OvCa from other metastasizing epithelial-derived cancers. The enhanced permeability of the tumor vasculature allows for the accumulation of proteins, including growth factors, cells, and fluid, and provides a rich tumor-promoting microenvironment. Thus, OvCa represents a disease of hyperpermeability, angiogenesis, cellular proliferation, and metastasis.

It has been postulated that the PTP4A phosphatases have a nodal role in cancer cell growth, survival, metabolism, migration, and invasion [17, 18]. In endothelial cells, increased PTP4A3 expression has been documented to enhance migration, tube formation, and angiogenesis [13, 14]. PTP4A phosphatases interact with a variety of protein partners, including cell surface receptors, intracellular signaling effectors, actin skeleton proteins, and ion channels [17]. Despite the relatively low *in vitro* catalytic activity of PTP4A with artificial substrates, the catalytic domain is functionally or structurally relevant in oncogenesis because phosphatase-deficient mutant proteins are unable to form tumors [34]. The generation of a PTP4A-targeted pharmacological small molecule tool set should help to uncover the mechanism by which PTP4A phosphatases alter cancer cell biology.

JMS-053 is a unique, potent, and selective small molecule antagonist of PTP4A3 *in vitro* phosphatase activity that suppresses the migration and decreases the viability of OvCa cells, and impedes the *in vivo* growth of OvCa (*i.e*., HeyA8-MDR) xenografts. Our data are consistent with JMS-053-mediated inhibition of RhoA, a previously described effector in the PTP4A3 signaling pathway [10, 12, 35]. Mechanistically, PTP4A1 has also been shown to regulate RhoA activation by binding and inhibiting the catalytic activity of p115 RhoGAP [9]. The binding interaction has been assigned to amino acids 30-120 of PTP4A1, which is 80% identical in PTP4A3 and contains the catalytic site. Moreover, the proposed binding sites for JMS-053 overlaps with the p115 RhoGAP binding site.

The pharmacological phenocopying of effects observed in PTP4A3 genetic knockdown models by JMS-053 fortifies the hypothesis that PTP4A3 is a viable molecular target for OvCa treatment [11, 13]. JMS-053 exposure also restored compromised barrier function after VEGF or LPS challenge in MVECs, and inhibited RhoA activation and sustained active Rac1, a pharmacodynamic endpoint of undamaged membrane permeability. Complementing these results, a structurally distinct small molecule inhibitor of PTP4A trimerization [7] also restored compromised barrier function.

RhoA GTPase has well defined roles in the regulation of cytoskeletal dynamics. These, in turn, impact cell cycle progression and proliferation, migration, invasion- and metastasis, which are all key aspects of cancer initiation and progression (reviewed in [32, 36]). Thus, targeting PTP4A3, upstream of RhoA, can have multi-faceted anti-tumor and anti-metastatic effects. To a large extent, the endothelial barrier integrity is regulated by the small GTPases, Rac1, and RhoA [37], which cycle between inactive, GDP-, and active, GTP-bound configurations. This cycle is regulated by GTPase activating proteins, which increase the intrinsic rate of GTP hydrolysis and guanine nucleotide exchange factors, which in turn promote GTPases into the GTP-bound state [38]. Rac1 and RhoA exert opposing effects on endothelial barrier function by inducing different patterns of cytoskeletal and cellular contact remodeling [39], often leading to either endothelial barrier protection (Rac1) or to endothelial barrier dysfunction (RhoA) [40]. Activation of RhoA by inflammatory mediators, including LPS, activates ROCK1/2, which consequently phosphorylates myosin light chain kinase and leads to actomyosin contraction, actin stress fiber formation, and disruption of endothelial barrier integrity (reviewed in [41]). It is notable that RhoA mediates the activation/phosphorylation of the ezrin/radixin/moesin proteins [42]. Ezrin is a putative phospho-substrate of PTP4A3, suggesting that PTP4A3 may not directly dephosphorylate ezrin; rather it may inhibit ezrin phosphorylation indirectly via down-modulation of RhoA-ROCK activity [43]. Similarly, inhibition of PTP4A3 may also prevent myosin light chain phosphorylation indirectly via RhoA inhibition. Moreover, with small G-proteins, including RhoA, the rate of nucleotide exchange is regulated by nucleotide-bound magnesium, which regulates the biological activity of the GTPase [44]. The magnesium-based regulation of RhoA is particularly relevant given recent work suggesting that PTP4A phosphatases bind the magnesium transporter cyclin M/ancient conserved domain protein (CNNM3) via the PTP4A phosphatase active site, thereby regulating intracellular magnesium transport [45].

In summary, pharmacological tools provide another valuable method to dissect the role of domains in enzymes and provide an orthogonal approach to genetic methods. We believe that our compound, JMS-053, along with other small molecules that have recently been described [7], will likely be useful in testing the hypothesis that the pharmacological inhibition of PTP4A3 can suppress OvCa by targeting both the tumor cell directly and endothelial cell hyperpermeability, which enables the infiltration of cells and soluble proteins into the tumor microenvironment and results in an accumulation of ascites fluid.

## MATERIALS AND METHODS

### Compounds and reagents

Thienopyridone, Compound 43, JMS-053, and JMS-038 (Figure 2A) were synthesized as previously described [7, 19]. DiFMUP was purchased from ThermoFisher Scientific (Waltham, MA). Dimethyl sulfoxide (DMSO) was obtained from VWR (Radnor, PA). All other reagents were obtained from Sigma-Aldrich unless otherwise indicated.

### Ovarian cancer cell lines

HeyA8, HeyA8-MDR, A2870IP2 (referred to as A2870 throughout), A2780CP20, COV362, SKOV3, SKOV3IP1, SKOV3TRIP2, and HI0-180 cells were obtained from Dr. Anil Sood (MD Anderson Cancer Center, Houston, TX) and have been described previously [46]. COV362 were obtained from Sigma-Aldrich (St. Louis, MO). All cell lines were authenticated by short tandem repeat DNA profiling prior to experimental studies. OVCAR4 cells were obtained from Charles River Laboratories (New York, NY). All cells were maintained in RPMI (Life Technologies, Grand Island, NY) supplemented with 10% fetal bovine serum (FBS), with no antibiotics, and were passaged <20 times.

### The Cancer Genome Atlas datamining

The ovarian serous cystadenocarcinoma dataset was accessed and mined through the Cancer Genome Atlas (TCGA) Research Network (Provisional 2017) (http://cancergenome.nih.gov/). Tumor samples with corresponding RNAseq (307 patients) and U133 microarray mRNA data (535 patients) were used for subsequent analysis.

### Immunohistochemical staining and scoring of patient OvCa tumors

Fifty-seven high-grade serous ovarian cancer specimens were obtained after surgical resection and prior to radiation or chemotherapy. Formalin-fixed paraffin embedded patient samples were tissue sectioned, and immunohistochemical stained for PTP4A3 by the University of Virginia Biorepository and Tissue Research Facility Core following standard procedures. Tissue and tumor sections were stained with anti-PTP4A3 (5 µg/mL, Abcam (ab82568), Cambridge, MA), which was previously used with human breast cancer [47], and antigen retrieval was by High hP TR Flex solution (Dako) at 97°C for 20 min. The immunogen for the PTP4A3 antibody was the first 100 amino acids of the protein, which are not identical to the first 100 amino acids in PTP4A1 and PTP4A2. It is assumed that this amino acid difference precludes cross reactivity with PTP4A1 and PTP4A2, but we cannot exclude that possibility. Tumor morphological classification was performed in accordance with the American Joint Committee on Cancer staging system. The study was approved by the Institutional Human Subjects Protection Review Board at the University of Virginia and carried out in accordance with their standards.

### *In vitro* biochemical analysis of JMS-053 and thienopyridone

PTP4A3 activity assays were performed in triplicate in 384 well Greiner bio-one black microtiter plates as previously described [19] with recombinant His_6_-tagged PTP4A3 and substrate DiFMUP (15 µM) incubated at 25°C for 30 min in 40 mM Tris-HCl (pH 7.0), 75 mM NaCl, 2 mM EDTA, and 4 mM DTT buffer. Reversibility assays were performed in a 100 μL total volume using the same assay conditions. His_6_-tagged PTP4A3 (1.25 μg) was pre-incubated for 30 min with 10X the IC_50_ of JMS-053 (180 nM) and then diluted to 1X IC_50_ (18 nM). Reactions were initiated with the addition of 45 µL of substrate for a final DiFMUP concentration of 4 μM (K_m_). Phosphatase activity was expressed as a percent maximal activity. To determine the competitive nature of the inhibition, we used a previously described assay [19] with fixed concentrations of the compounds and DiFMUP substrate concentrations that were varied from 2.5-25 μM and the data were analyzed with GraphPad Prism version 7.00 (GraphPad Software, La Jolla CA).

### Phosphatase specificity of JMS-053

The inhibition specificity of JMS-053 was determined for 26 phosphatases by Eurofins (Dundee, Scotland, UK) with their PhosphataseProfiler™ platform using 10 µM DiFMUP as a substrate and fluorescent intensity at A360_EX_/A450_EM_. Inhibitory effects were calculated as the mean percent phosphatase activity remaining compared with the activity of the vehicle control. Kinase profiling of JMS-053 was performed by Luceome Biotechnologies (Tucson, AZ) using their luciferase-based KinaseSeeker™ platform. JMS-053 was evaluated at 1 µM in duplicate in both profiling platforms. The CDC25B assays were performed with a slight modification to our previously described methods [48] using recombinant enzyme and the substrate DiFMUP at ∼3X the K_m_ (150 µM). PTP4A1 and PTP4A2 assays were performed in triplicate in 384 well microtiter plates using the same conditions as for PTP4A3 as indicated above. PP2A was a gift from Professor David Brautigan (University of Virginia) and was assayed with the substrate DiFMUP at ∼3X K_M_ (150 µM) in 120 mM Tris-HCl (pH 7.0), 225 mM NaCl, 0.1% BME, and 0.1% BSA buffer. All assays were incubated for 30 min at 25°C. Fluorescence was measured using a SpectraMax M5 plate reader (Molecular Devices, LLC, Sunnyvale, CA) at A358_EX_/A455_EM_. The human active site phosphatase phylogenetic tree comprising 14 amino acids (XXXX(His)(Cys)XX(Gly)XX(Arg)XX, where X is any amino acid) was modeled on the published [49] dendrogram supplemented with the alignment of other PTPs tested using ClustaW2 and the full length amino acid sequence trees were modeled using the longest isoform [50].

### Computational studies

Drug-like properties for the compounds were determined with Data Warrior (http://www.openmolecules.org/datawarrior/). Chemical similarity analyses were performed with PubChem (https://pubchem.ncbi.nlm.nih.gov) and ChemSpider (www.chemspider.com) using a 2D Tanimoto score of ≥0.8. Modeling software Maestro version v11 (Schrödinger, LLC, New York, NY), and Insight 2005 and Discovery Studio v16 (both: Dassault Systèmes BIOVIA, San Diego, CA), operating on a Dell (Round Rock, TX) Precision T7600 with Red Hat (Raleigh, NC) Enterprise Linux 6.7, were used for all modeling studies. The three-dimensional (3D) coordinates of PTP4A3 were extracted from PDB entry 5TSR [22]; the 3D coordinates of inhibitor JMS-053 were obtained from the Cambridge Crystallographic Data Centre (CCDC number 1476250) [19]. The structure of PTP4A3 was energy refined in Insight 2005 using a previously reported strategy starting with hydrogens only minimization, followed by side-chain relaxation, and finally, all atoms optimization until the norm of the gradient was <0.001 kcal/Å [51]. The RMSD of the energy refined structure *versus* the starting X-ray structure was 1.29 Å, which was well within crystallographic resolution. Maestro and Discovery Studio binding site detectors were used to identify a location for JMS-053 binding in a cavity (volume ∼ 350 Å^2^) surrounded by helices α3, 4, 6 and loop residues 68 - 78. This cavity was chosen for docking studies given that its location was removed from the active site, yet in contact with the critical WPD loop. Docking of the inhibitor in this site was guided by the constant scoring of favorable and unfavorable intermolecular, atom-atom contacts using the HINT program (implemented in Discovery Studio) as described previously [51]. Briefly, an systematic optimization of the binding pose applied iterative steps and manual adjustments to the inhibitor in Cartesian space, as well as to inhibitor bond torsions, in conjunction with adjustments to amino acid side chain torsions. The Maestro refine loops algorithm was applied to loop residues 68 - 78, followed by all atoms minimization of the complex using conjugate gradients in Insight 2005 until the norm of the gradient was < 0.001 kcal/Å.

### Thermal shift biophysical validation of JMS-053 binding

PTP4A3 melting curves were obtained at a protein concentration of 16 μM (50 mM Tris 150 mM NaCl, 1 mM DTT pH 7.4) with 20X SYPRO orange (Invitrogen) and a ligand concentration of 100 μM. Fluorescence was monitored using a QuantStudio 6 Flex real-time PCR system (Applied Biosystems). Scans were measured from 25°C to 65°C at a scanning rate of 1°C/min. The data were analyzed using Derivative methods to assess the melting temperature (*T*_*m*_) using Graphpad Prism as previously described [52].

### Real-time quantitative polymerase chain reaction (RT-qPCR)

Total RNA was purified from HeyA8 cells using an RNAeasy Plus RNA isolation kit per the manufacturer’s protocol (Qiagen, Valencia, CA). A total of 500 ng of mRNA were converted to cDNA using the RT^2^ first strand synthesis kit (Qiagen). Primers were obtained from Qiagen and PTP4A3 amplification was performed at a final primer concentration of 400 nM. Real-time monitoring of the qPCR reaction was performed on a BioRad CFX Connect thermocycler with RT^2^ SYBR Green ROX Mastermix (Qiagen). The program was run at 95°C for 10 min, and 40 cycles at 95°C for 15 sec and 60°C for one min. PTP4A3 gene expression was normalized to human GAPDH and β-actin.

### Two-dimensional (2D) viability assay

Compounds were tested for their effects on viability using OvCa cell lines and a resazurin-based CellTiter Blue (Promega, Fitchburg, WI) 2D assay system with previously described procedures [53]. All OvCa cell lines were cultured in complete growth medium with 500 cells/22 μL being seeded into each well of a 384 well black/clear microtiter plate (VWR). Compounds (3 μL) were added immediately to produce a final DMSO concentration of 0.5% matching the vehicle control final DMSO concentration. The positive control wells had a final concentration of 10% DMSO. The microtiter plates were incubated for 44 h (37°C, 5% CO_2_) at which time 5 μL of the resazurin-based CellTiter Blue reagent were added. Following an additional 4 h of incubation (37°C, 5% CO_2_), data were captured on a SpectraMax M5 multimode plate reader with an *A*560_EX_/*A*590_EM_. IC_50_ values were calculated using GraphPad Prism 7.0 software.

### Spheroid growth and formation assays

Exponentially growing OvCa cells were harvested and seeded (100 cells/22 µL for HeyA8, HeyA8-MDR and HI0-180, and 250 cells/22 µL for OVCAR-4, A2780, A2780CP20, SKOV3, SKOV3IP1, SKOV3TRIP2, and COV362) in each well of a 384 well ultralow attachment U-bottom microplate (Corning, Corning, NY). Plates were incubated for 24 h (37°C at 5% CO_2_) to allow for spheroid formation. Compounds (3 μL) were then added in final DMSO concentrations of 0.5%, as was the vehicle control (0.5% DMSO). The positive control wells contained 10% DMSO. The microtiter plates were incubated for 48 h (37°C, 5% CO_2_) and 25 μL of the CellTiterGlo 3D reagent (Promega, Fitchburg, WI) was added. Plates were incubated with shaking for 30 min at room temperature. Luminescence data were captured on a SpectraMax M5 multimode plate reader. JMS-053 effects on OvCa spheroid size were determined using a PerkinElmer Operetta CLS high content image analysis system (PerkinElmer, Waltham, MA). Exponentially growing HeyA8 cells were harvested, seeded (100 cells/22 µL), incubated, and treated with compound as described above. Brightfield spheroid images were captured 48 h after JMS-053 or JMS-038 exposure using a 10X objective. Spheroid area was quantified using Harmony software (PerkinElmer). Data were analyzed and IC_50_ values calculated using GraphPad 7.0.

### Migration assay

OvCa cell migration was quantified using our previously described *in vitro* scratch wound healing assay [13]. For compound treatment studies, HeyA8 cells were grown to confluent monolayers in 24 well tissue culture plates (BD Biosciences, San Jose, CA). Each well (*n* = 3 per compound or control treatment) was scratched longitudinally with a pipette tip and incubated for 14 or 15 h to enable gap closure. Images of the cell migration front were captured and the migration area was determined by measuring the distance between cell fronts at multiple points in the images using ImageJ Fiji software and then calculating the open area remaining from the scratch.

For shRNA studies, HeyA8 cells were seeded at 5 x 10^5^ cells per well of a six well tissue culture dish. At 90% confluency, cells were forward transfected with 2.5 µg scrambled or PTP4A3-targeted pGFP-C-shLentiRNA encoding plasmids (Origene Technologies, Rockville, MA) complexed with lipofectamine 3000 (1:3) (ThermoFisher). After 16 h, culture medium was replaced. Cells were harvested by trypsin treatment at 32 h post-transfection and reseeded in 12 well microtiter plates at 2 x 10^5^ cells/well and grown to confluency. The scratch wound assay was carried out as described above.

### Measurement of endothelial barrier function

The barrier function of endothelial cell monolayers grown on electrode arrays was estimated by our previously described [26, 27] electric cell-substrate impedance sensing (ECIS) method using an ECIS model 1600R from Applied BioPhysics (Troy, NY). Experiments were conducted on cells seeded at a density of 6 x 10^4^ cells/well in 8W10E+ arrays that achieved at least 800Ω baseline steady-state resistance and capacitance between 22-29 nanofarads at a frequency of 4000Hz. Each 0.6 mL well of the 8 well arrays had 40 small gold film surface active electrodes (2 mm^2^ area) and a large counter electrode. A uniform confluent endothelial monolayer reduced the amount of current flowing across the gold electrodes to the counter electrode, and thus increased the resistance. The transendothelial electrical resistance (TEER) measured dynamically across the monolayer reflected the combined resistance between the ventral surface of the cell and the electrode, reflective of focal adhesion, as well as the resistance between cells. Intercellular gaps increase current flow and reduce resistance. Thus, a change in TEER represented a change in cell-cell adhesion and/or cell-matrix adhesion. All experiments used primary human lung microvascular endothelial cells (MVEC) that were harvested, identified, and cultured in-house from fresh specimens obtained from patients undergoing pneumonectomy or lobectomy, as previously described [54] and in accordance with our Institutional Review Board criteria. MVEC were exposed to 100 ng/mL VEGF, 0.5 endotoxin units (EU) LPS, or vehicle control. In some studies cells were pre-treated and then continuously treated with 5 μM of JMS-053, JMS-038, or Compound 43. In other studies, cells were treated 3 h after exposure to VEGF or LPS. Experiments were performed in quadruplicate and data were expressed as mean values ± SE. One-way or two-way ANOVA with Bonferroni post-hoc test was performed to determine statistically significant differences among groups (*p* < 0.05).

### RhoGTPases activity assay

RhoA activation in HeyA8 cells was detected by the luminescence formatted RhoA G-LISA Activation Assay (Cytoskeleton Inc., Denver, CO), according to manufacturer’s instructions. Following a 24 h serum starvation in RPMI supplemented with 0.1% fatty-acid free bovine serum albumin, HeyA8 cells at ∼60% confluence were pretreated in serum-free medium for 30 min with compounds in a final DMSO concentration of 0.5%, as was the vehicle control. HeyA8 cells were then stimulated for 30 min with RPMI containing 10% FBS and the compounds in a final DMSO concentration of 0.5% and harvested on ice. Total protein was determined using Precision Red Advanced protein assay reagent (Cytoskeleton Inc.). Fifteen µg of cell lysate protein per Rho-GTP affinity well were incubated at 4°C for 30 min on an orbital shaker to facilitate binding. As a positive control, 1 ng of purified recombinant RhoA was bound to the affinity well. The provided primary and secondary antibodies were used at a 1:250 and 1:500 dilution, respectively. Luminescence data were captured on a SpectraMax M5 multimode plate reader set to 50 ms integration time following 2 min incubation with luminescence detection reagent.

Human MVEC were pretreated with vehicle (DMSO) or JMS-053 (5 µM) prior to vehicle or LPS treatment (0.5 EU/mL) for 1 or 24 h. MVEC RhoA activation was detected using the bead pull-down format RhoA Activation Assay Biochem Kit (Cytoskeleton Inc.). Cells were lysed in CelLyticM Lysis Reagent and total protein in the lysate was determined with a Pierce BCA protein assay (ThermoFisher Scientific). Five hundred µg of total cell lysate protein were incubated at 4°C with 80 µg Rhotekin-RBD protein beads for 1 h rotating. After precipitation, the complexes were washed four times with the wash buffer, eluted in 2X SDS-PAGE sample buffer, immunoblotted, and probed with RhoA antibody. GTP-bound RhoA detection was performed using a monoclonal rabbit RhoA antibody (Cell Signaling, no. 2117) [26]. Proteins were visualized using a LICOR Odyssey CLx imaging system (Lincoln, NE). Aliquots were taken from supernatants prior to precipitation and were used to quantify total RhoA. Rac1 activity was determined using the Rac Activation Assay Biochem Kit (Cytoskeleton Inc., BK-035) per the manufacturer’s instructions. Secondary mouse and rabbit antibodies were purchased from LICOR. ImageJ software was used to perform densitometry of immunoblots. All data were expressed as mean values ± SE. Student’s *t*-test or one-way or two-way ANOVA with Bonferroni post hoc test was performed to determine statistically significant differences among groups (*p* < 0.05).

### *In vivo* evaluation of JMS-053

Pharmacokinetic studies were conducted by Sai Life Sciences Ltd (Hinjewadi, India) using 20% 1-methyl-2-pyrrolididone, 25% Kolliphor HS 15, and 55% 1X phosphate buffered saline as the vehicle and a single IP dose of 10 mg/kg JMS-053. Blood samples were collected under light isoflurane anesthesia from retro orbital plexus pre-dose and at eight time points, the last being at 24 h. Three mice were used for each time point. Sample were processed for analysis by protein precipitation using acetonitrile and analyzed by liquid chromatography/mass spectroscopy/mass spectroscopy.

For *in vivo* antitumor experiments, HeyA8-MDR cells in exponential growth phase were detached from the monolayer with 0.25% trypsin and 20 µg/mL EDTA in PBS and resuspended in 10% FBS-containing RPMI, pelleted, and resuspended in serum-free RPMI at a concentration of 5 x 10^6^ cells/mL. Female athymic nude mice (Envigo, Dublin, VA) were injected intraperitoneally (IP) (200 µL/injection) with cells and after one week mice (n = 10 per group) were randomized for treatment with vehicle (20% 1-methyl-2-pyrrolididone, 25% Kolliphor HS 15, and 55% 1X phosphate buffered saline) or with vehicle plus 10 mg/kg JMS-053. Mice were treated IP daily (100 µL/ injection) for 5 days with a 2-day holiday followed by 4 additional days of treatment before being sacrificed. All tumors were excised and weighed. All *in vivo* procedures were performed using the University of Virginia approved IACUC protocols. Animal care was administered in accordance to guidelines established by the American Association for Accreditation of Laboratory Animal Care.

### Statistical analysis

All statistical analyses were performed with GraphPad Prism 7.0. Data are presented as average (mean) ± SD or SE. P values were calculated with Student’s *t* test for comparisons involving two groups or one-way or two-way ANOVA for comparisons involving >two groups. *P* < 0.05 was considered statistically significant. Each experiment is represented by at least three biological replicates and three technical replicates (per independent experiment).

## SUPPLEMENTARY MATERIALS FIGURES AND TABLES


