# Supplementary material for: Targeting ovarian cancer and endothelium with an allosteric PTP4A3 phosphatase inhibitor
Source: Oncotarget. 2017 Dec 30;9(9):8223–40. doi: 10.18632/oncotarget.23787 (PMC5823565; doi:10.18632/oncotarget.23787)
Supplement: Supplementary file 1 [file oncotarget-09-8223-s001.pdf]

Targeting ovarian cancer and endothelium with an allosteric PTP4A3 phosphatase inhibitor

SUPPLEMENTARY MATERIALS

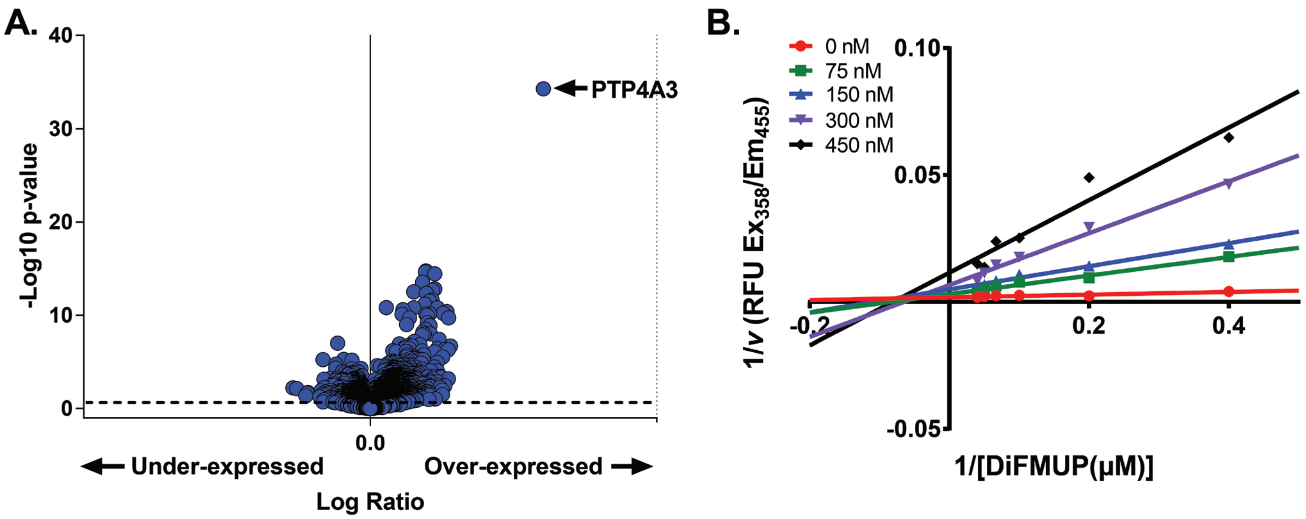

Supplementary Figure 1: U133 microarray analysis confirmed PTP4A3 mRNA was the most abundant mRNA in the OvCa patient TCGA dataset and *in vitro* kinetic studies revealed thienopyridone is a noncompetitive inhibitor. Panel A. Similar to RNAseq data, PTP4A3 mRNA was the most abundant mRNA in TCGA OvCa patients as determined by U133 microarray (535 OvCa patients). Panel **B**. Lineweaver Burk plot indicated noncompetitive inhibition by thienopyridone against PTP4A3.

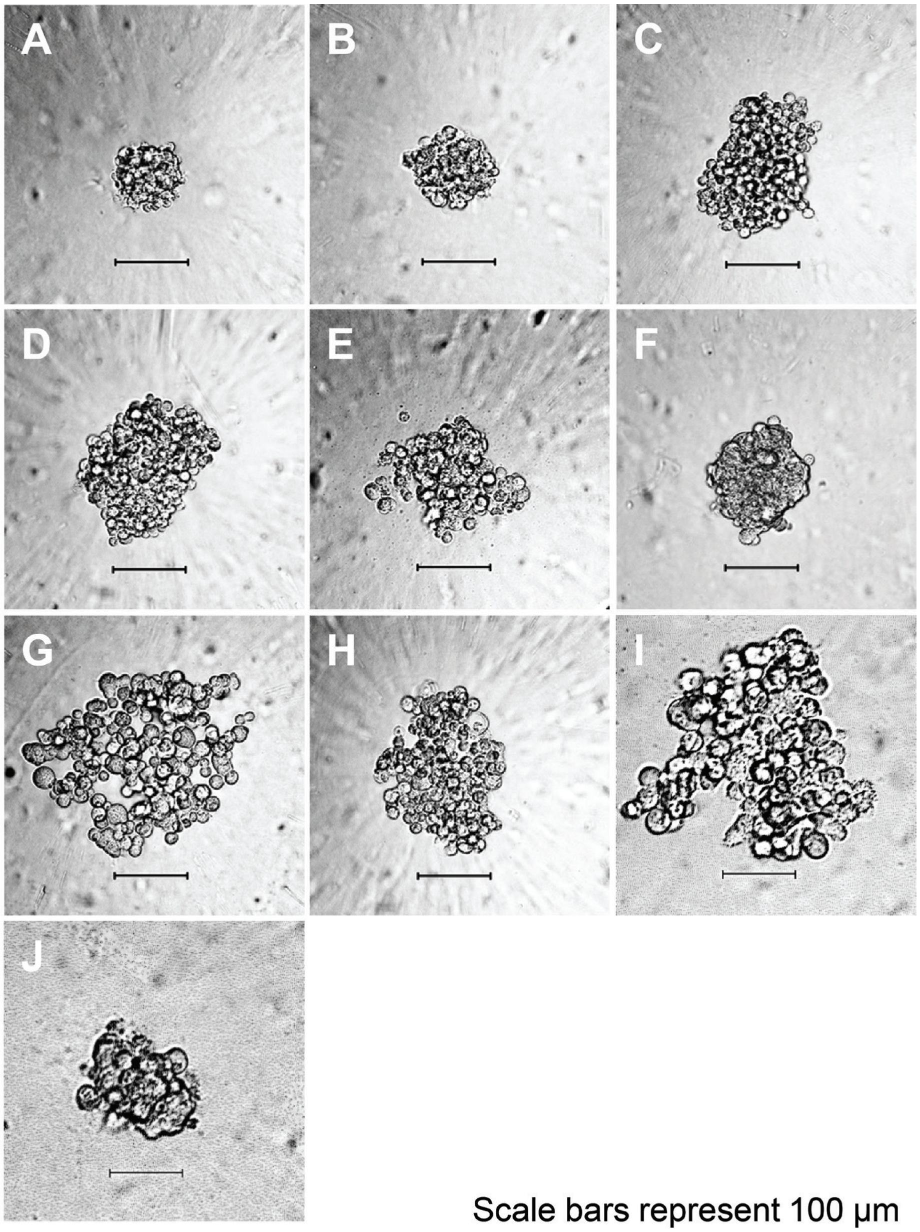

Supplementary Figure 2: Variation in OvCa cell spheroid morphology. Untreated OvCa cell lines exhibit widely varied spheroids morphology ranging from tight circular to loosely aggregated. Panel **A**. HeyA8, tight circular; Panel **B**. HeyA8-MDR, tight circular; Panel **C**. A2780, moderately circular; Panel **D**. A2780CP20, moderately circular; Panel **E**. OVCAR4, loosely circular; Panel **F**. SKOV3, loosely circular; Panel **G**. SKOV3IP1, loosely aggregated; Panel H. SKOV3TRIP2, loosely aggregated; Panel **I**. COV362, loosely aggregated; Panel J, COV362-resistant, moderately circular. Cells were seeded as described in Materials and Methods. Images were acquired 24 h after cell seeding. Bar = 100 µm.

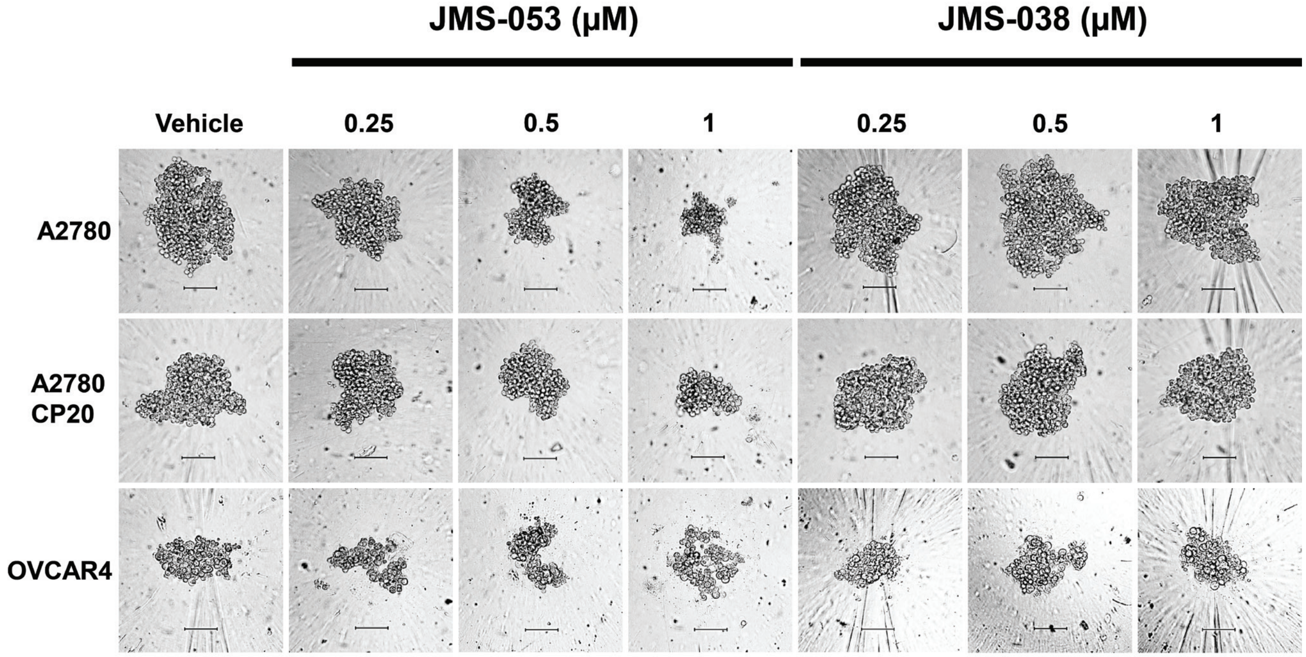

Supplementary Figure 3: Effect of JMS-053 treatment on OvCa spheroid growth. A2780, A2780CP20 and OVCAR-4 cells were seeded as described in Materials and Methods. Twenty-four h later, the resulting spheroids were exposed to vehicle, JMS-053 (0.25-1 μM) or JMS-038 (0.25-1 μM) and images were acquired 24 h later. Bar = 100 μm.

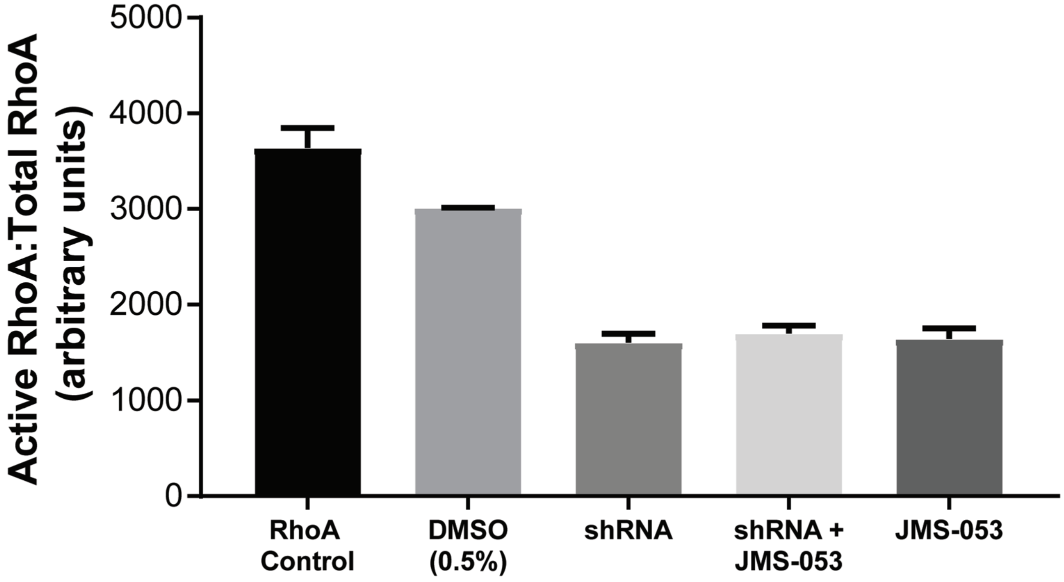

Supplementary Figure 4: JMS-053 inhibits RhoA activation in HeyA8 cells via PTP4A3. HeyA8 cells were treated with PTP4A3-targeted shRNA for 24 h in complete medium. Cells were starved in serum-free RPMI 1640 for 24 h, then activation of RhoA was induced by exposure to 10% serum. Cells were pre-treated with 500 nM JMS-053 or vehicle control (0.5% DMSO) for 30 min prior to serum stimulation and for 30 min while being stimulated with medium containing 10% FBS. GTP bound RhoA in equal amounts of total lysate was quantified by G-LISA RhoA Activation assay. Constitutively active RhoA was used as a positive control for binding of GTP-RhoA to G-LISA. The data were expressed in arbitrary units relative to total RhoA present in cells with the mean ± SEM (N=2).

Supplementary Table 1: Predicted drug-like properties of JMS-053. The drug-like properties of JMS-053 were determined using Data Warrior.

Properties	Thienopyridone	JMS-053
H Donor	2	2
H Acceptor	3	4
Total Surface Area	177.9	182.0
Relative Polar Surface Area	0.338	0.402
Polar Surface Area	83.36	98.26
cLogP	2.125	1.545
cLogS	-4.283	-3.430
Druglikeness	3.683	4.393

Supplementary Table 2: Profiled of JMS-053 for inhibition against 50 kinases.

Kinase	Family	Activity Ave ± SD		Kinase	Family	Activity Ave ± SD
AAK1	Other	82 ± 1		MARK2	CAMK	86 ± 3
ABL1	TK	95 ± 3		MLK2	TKL	101 ± 3
ACVR2A	TKL	93 ± 2		MLK3	TKL	101 ± 3
AKT1	AGC	87 ± 0		MSK2	AGC	94 ± 2
AKT2	AGC	87 ± 5		MST2	STE	99 ± 5
AKT3	AGC	91 ± 3		MUSK	TK	98 ± 5
AMPKa1	CAMK	98 ± 1		p38a	CMGC	50 ± 3
AURKA	Other	104 ± 1		PDGFRB	TK	87 ± 0
AURKB	Other	90 ± 2		PDPK1	AGC	92 ± 2
AURKC	Other	90 ± 0		PIM1	CAMK	86 ± 1
CAMK1D	CAMK	90 ± 2		PKA	AGC	87 ± 1
CAMK2D	CAMK	100 ± 4		PKCe	AGC	86 ± 1
CDK5	CMGC	84 ± 2		PKG1	AGC	98 ± 2
CLK2	CMGC	76 ± 2		PLK4	Other	90 ± 3
DAPK3	CAMK	86 ± 1		PRKX	AGC	94 ± 1
DDR1	TK	88 ± 3		PYK2	TK	91 ± 1
EPHA5	TK	96 ± 1		RSK2	AGC	100 ± 2
EPHB2	TK	98 ± 8		SLK	STE	85 ± 0
FLT1	TK	90 ± 2		SNF1LK	CAMK	100 ± 2
FLT3	TK	108 ± 2		SNF1LK2	CAMK	92 ± 1

HCK	TK	93 ± 1		SRC	TK	83 ± 6
IGF1R	TK	87 ± 0		TNK1	TK	84 ± 1
ITK	TK	87 ± 2		VEGFR2	TK	90 ± 0
KIT-KD	TK	94 ± 1		YANK2	AGC	88 ± 3
MARK1	CAMK	87 ± 3		YSK1	STE	100 ± 2
